# Hairless mutation: a driving force of humanization from a human–ape common ancestor by enforcing upright walking while holding a baby with both hands

**DOI:** 10.1111/j.1365-2443.2012.01592.x

**Published:** 2012-04

**Authors:** Shizuyo Sutou

**Affiliations:** School of Pharmacy, Shujitsu University1-6-1 Nishigawara, Naka-ku, Okayama 703-8516, Japan

## Abstract

Three major characteristics distinguish humans from other primates: bipedality, practical nakedness, and the family as a social unit. A hairless mutation introduced into the chimpanzee/human last common ancestor (CLCA) 6 million years ago (Mya) diverged hairless human and hairy chimpanzee lineages. All primates except humans can carry their babies without using their hands. A hairless mother would be forced to stand and walk upright. Her activities would be markedly limited. The male partner would have to collect food and carry it to her by hand to keep her and their baby from starving; irresponsible and selfish males could not have left their offspring. The mother would have sexually accepted her partner at any time as a reward for food. Sexual relations irrespective of estrus cycles might have strengthened the pair bond. Molecular and paleontological dating indicates that CLCA existed 6 Mya, and early hominin fossils show that they were bipeds, indicating that humanization from CLCA occurred rapidly. A single mutation in animals with scalp hair is known to induce hairless phenotype (ectodermal dysplasia). Bipedalism and hairlessness are disadvantageous traits; only those who could survive trials and tribulations in cooperation with family members must have been able to evolve as humans.

## Introduction

Several characteristics separate humans from other primates: bipedality, hairlessness, a family as a social unit, a large neocortex, small canine teeth, uses of tools, fire, and language, culture and civilization, and so forth. Especially, the first three are considered to constitute basic key factors of the origin of humans. Other important characteristics such as the use of tools and fire are considered to have achieved after the establishment of bipedalism, which liberated hands from walking. Language seems to have emerged long after bipedalism, which allowed the brain grow larger at the *Homo* stage.

The origin of bipedality has remained a matter of interest for over a century. Many hypotheses and ideas have been put forward to explain bipedalism. Use of tools compelled hominins to stand upright (Washburn 1960). Upright posture and bipedal gait were useful for vigilance against predators (Day 1986). Bipedality might have evolved for food transport in a dry savannah habitat (Hewes 1961). A terrestrial feeding posture, rather than walking adaptation, was important for bipedalism (Jolly 1970). Bipedal threat display and appeasement behavior were important for the peaceful resolution of intragroup and interspecific conflicts (Jablonski & Chaplin 1993). Upright hominins were able to forage longer in the open sun (Wrangham 1980). Hominins were scavengers, and bipedalism was a necessary adaptation that enabled migration for sufficient food by scavenging (Sinclair *et al.* 1986). When biped hominins moved to an open savannah environment, they had a thermoregulatory advantage (Wheeler 1991). Analyses of walking energetics and biomechanics have showed that bipedalism in early, ape-like hominins could have been less costly than quadrupedal knuckle-walking (Sockol *et al.* 2007). Provisioning must be critical for upright walking (Lovejoy 1981). Other theories include some combination of them or others. A brief and limited survey of reports in the literature shows varied hypotheses or ideas, but none is conclusive.

Hairlessness distinguishes humans from other primates. Lack of availability of skin or hair fossils must be the major reason for the scarcity of hypotheses and ideas about human nakedness in comparison with those for bipedalism. Nonetheless, several have been proposed. Earlier, T. Huxley dealt intensively with nakedness. ‘His conclusion was that the loss of body hair was due to sexual selection: that men (or more specifically, he implies, women) became hairless to attract a mate’ (Morgan 2009). If so, then why did at least a few other primates not become naked via sexual selection? The body-cooling hypothesis was apparently most persuasive to explain human hairlessness. The reduction in body hair provided a thermoregulatory advantage to hominins with a large brain, which is vulnerable to thermal damage. According to this hypothesis, bipedality preceded body hair reduction (Wheeler 1984, 1985). Intuitively, naked skin can be regarded as disadvantageous at lower ambient temperatures. Indeed, the body-cooling hypothesis has been criticized that ‘the results do not indicate that the initial step in the denudation process occurred in open hot environments, nor that bipedality preceded body-hair reduction’ (do Amaral 1996). Another hypothesis is that humans, who were able to regulate their environment using fire, shelters, and clothing, shed their fur to rid their bodies of disease-spreading ectoparasites such as lice (Pagel & Bodmer 2003). This is inapplicable to early hominins, who had no ability to use fire, shelter, or clothing. Indeed, a molecular clock analysis shows that human body lice originated approxiamately 72 000 years ago and suggests that clothing is a recent innovation in human evolution (Kittler *et al.* 2003). Here, again, none is conclusive as to the origin of human nakedness.

The reproductive unit of humans is a family, which constitutes the basic unit of society and which clearly distinguishes humans from other primates. The social organization of orangutans is a loose community. A typical reproductive unit consists of one male and one or more female clusters (Singleton & van Schaik 2002). Single male and multimale groups exist among gorillas. Genomic analyses show that the dominant silverback in a multimale group does not always monopolize females within his group (Bradley *et al.* 2005). Chimpanzees form a closed reproductive unit consisting usually of 20–80 males and females who are promiscuous. Genomic analyses show that 7% offspring in a West Africa group were sired by extra-group males (Vigilant *et al.* 2001); extra-group paternity reached 50% among Tai Forest (Cote d’Ivoire) chimpanzees (Gagneux *et al.* 1999). Consequently, the monogynous nature of a human family is unique, suggesting that the origin of the family is intrinsically associated with the origin of humans. Their unique sexual and reproductive human behavior (Lovejoy 1981) might be explained in the context of the unique family formation.

Consequently, why bipedalism, hairlessness, and the human family originated persist as puzzling questions despite the great efforts undertaken to explain them. Separation of the respective issues might be a cause of confusion: they might share the same root at the origin. Basic traits that define a clade must have remained as they were at the start. Observation of baby-raising primates, including humans, hints that hairlessness triggered bipedalism, family formation, and some other basic human characteristics. Here, a hairless mutation hypothesis is put forward to explain humanization from the chimpanzee/human last common ancestor (CLCA). The essence of this hypothesis was presented to the annual meeting of the Molecular Biology Society of Japan (Sutou 2010).

## Bipedal earlier hominins

It is commonly accepted that we *Homo sapiens* deviated from the common ancestor of humans and African apes, gorillas, and chimpanzees. Because chimpanzees are phylogenetically closer to humans than to gorillas, the use of CLCA is more relevant than the gorilla/human last common ancestor to determine the time to a most recent common ancestor (TMRCA), which is estimated to have existed between 5 and 7 million years (My). A very early fossil of the human lineage close to CLCA is *Sahelanthropus tchadensis*, which was found in the Djurab Desert, northern Chad (Vignaud *et al.* 2002). Associated faunas suggest that the fossils are 6–7 million years old and that the hominins lived close to a lake but not far from a sandy desert. Analysis of the basicranium suggests that *S. tchadensis* was an upright biped (Zollikofer *et al.* 2005). The femoral morphology of *Orrorin tugenensis* 6 million years ago (Mya) from Kenya exhibits bipedalism (Galik *et al.* 2004; Richmond & Jungers 2008). Another earlier species of *Ardipithecus kadabba* from the Middle Awash area of Ethiopia was dated to 5.2–5.8 Mya; it was associated with a wooded paleoenvironment. The proximal foot phalanx is consistent with bipedalism (Haile-Selassie 2001). Similarity of dentition of these three early hominins suggests the possibility that the three might be involved in a single genus (Haile-Selassie *et al.* 2004).

It had been suggested that the age of the three earlier hominin taxa (5.2–7 Mya) was followed by the age of later hominin taxa consisting of bipedal *Australopithecus* species (4 to ca. 1 Mya) represented by *Australopithecus anamensis* is and *Australopithecus afarensis* until the discovery of *Ardipithecus ramidus* (White *et al.* 1994). *Ardipithecus* might have lived ca. 6–4 Mya. ‘Ardi’ was an *A. ramidus* woman who lived in the Afar Rift region of northern Ethiopia 4.4 Mya (White *et al.* 2009b). She was found with most of her skull, pelvis, teeth, hands, feet, and other parts along with many other *A. ramidus* specimens. The extraordinarily well preserved and reconstructed skeleton of Ardi, together with a large collection of animal and plant fossils (more than 15 000) around her (Louchart *et al.* 2009; White *et al.* 2009a), provides reliable information related to early human evolution. She is considered to have stood approximately 120 cm tall and to have weighed 50 kg. Her face was small; her brain was also small (300–350 cm^3^), similar to that of a present bonobo or a present female chimpanzee. She and *A. ramidus* males had a reduced canine/premolar complex (Suwa *et al.* 2009) and showed no sexual dimorphism in body size (Lovejoy *et al.* 2009), suggesting less social aggression. She was a denizen of woodlands with small patches of forest, not of the open, grassy terrain, as animal and plant fossils around her show. She was probably omnivorous and ate nuts, insects, snails, and small animals found among the trees and on the ground, and did not feed much in the open grassland. Importantly, she was apparently a biped, showing that our ancestors walked upright before they evolved a larger brain. She had no characteristics of the suspension, vertical climbing, and knuckle-walking that present great apes have (Lovejoy *et al.* 2009).

These findings match well with an early presupposition that ‘the stem Hominidae were small creatures in which the trunk was specially adapted to various orthograde positional behaviors’ (Tuttle 1975). The findings also definitely negate the notion that inhabitation of grassland or open savannahs was the driving force of the origin of upright walking. Present great African apes cannot be the models of our ancestors. The skull and teeth of *A. ramidus* resemble those of *S. tchadensis*. In fact, *S. tchadensis*, *O. tugenensis,* and *A. kadabba* resemble one another overall (White *et al.* 2009b). They might belong to a single genus (Haile-Selassie *et al.* 2004). Their living periods overlap at least in part. These findings suggest that *A. ramidus* is the fourth member of the early hominin genus. Even if that is not so, Ardi must by and large represent images of our ancestors immediately after separation from CLCA. Consequently, hominins must have been bipedal from the very beginning.

## Not gradual modifications but sudden humanization from CLCA

Molecular data confirm the African apes as our closest living relatives and place the CLCA and modern humans recently, perhaps 5–7 Mya (Page & Goodman 2001). Other genetic evidence suggests that human speciation from CLCA occurred more recently than 6.3 Mya (Patterson *et al.* 2006). Genomic DNA sequencing data are now available. Human (International Human Genome Sequencing Consortium 2001; Venter *et al.* 2001) and chimpanzee (Chimpanzee Sequencing and Analysis Consortium 2005) genomes were deciphered; the genomic difference between the two is 1.2% (Cheng *et al.* 2005). Granted that base changes of 1% require 10 My, TMRCA is estimated as 6 My. Another molecular analysis using 36 nuclear genes indicates that TMRCA is 5.4 ± 1.1 My (Stauffer *et al.* 2001). It is of great interest to learn that divergence between chimpanzee and human lice was estimated to occur 5.6 Mya (Reed *et al.* 2004).

The salient implication is that the age of earlier hominins, *S. tchadensis*, *O. tugenensis*, and *A*. *kadabba* (5.2–7 Mya), is very close to that of CLCA. Overlapping or matching of paleontological and genetic ages indicates little or no intervening period between CLCA and the appearance of these earlier hominins. In other words, humanization in essence had not been achieved little by little after a long series of gradual modifications, such as deeply hairy to moderately hairy to slightly hairy. This is inconsistent with the idea that a large fraction of new species emerged from a single, rare, stochastic event (Venditti *et al.* 2010). Taken together, a hairless mutation could be regarded as a plausible cause for humanization.

## How do primates carry their babies?

**Figure 1:**
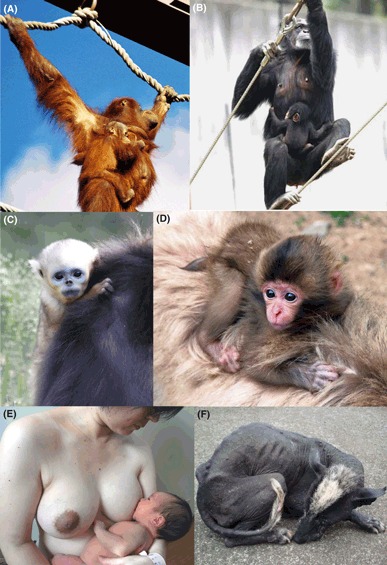
Baby/child-carrying hairy primates, a lactating human mother, and a hairless dog. All primate mothers excepting those of humans carry their young without using their hands, even when moving through the jungle tree canopy; hair is a baby carrier (A–D). A, a pair of sky-walking Bornean orangutans (*Pongo pygmaeus pygmaeus*) 17 m above the ground (courtesy of Ms H. Takahashi); (B) a pair of sky-walking chimpanzees (*Pan troglodytes*) 15 m above the ground (courtesy of Mr M. Nishizawa); (C) a pair of golden snub-nosed monkeys (*Rhinopithecus roxellana*) (courtesy of Ms M. Fukatsu); (D) a pair of Japanese macaque (*Macaca fuscata*) (courtesy of Mr T. Sakai); (E) a pair humans (*Homo sapiens*) (courtesy of Dr H. Shinozaki); and (F) a Mexican hairless dog (*Canis lupus familiaris*) (courtesy of the Mutsugoro Animal Kingdom). A semidominant mutation of a *FOXI3* family gene rids of most body hairs except for scalp and tail hairs.

Many visual and written records have been made of the behaviors of babies of monkeys and apes, most of which live in forests, woodlands, or jungles, and some of which, like baboons, live in open grasslands or savannahs. A newborn or infant baby clings to the mother by grasping her hair firmly, as although the baby was an appendage of the dam. Wherever they live, primate babies, except those of humans, are transported without dams’ support, even through the jungle tree canopy. The mothers can use their four limbs freely (Fig. 1A–D). In sharp contrast, a human baby has nothing to grasp; the mother must hold the baby with at least one hand or more safely with both hands (Fig. 1E). Consequently, the most fundamental and important difference between humans and other primates exists at birth: whether the babies themselves can cling to their mothers. What would have happened if a hairless mutation occurred in CLCA? Hairlessness must have compelled mothers to hold their babies with their hands, thereby depriving them of the use of their hands for quadrupedal climbing, walking, and running. Instead, it bestowed them with the opportunity and burden of walking upright. The hands that were required for baby-raising could then become used increasingly for collecting and carrying food, handling objects, manufacturing tools, throwing stones, and so forth as characteristic human behaviors. Consequently, hairlessness could have enhanced the division of labor of forelimbs with great dexterity and hind limbs with strong power for humanization, if it occurred once in CLCA.

## The family as a social unit enhanced by hairlessness

When CLCA lost their hair because of a hairless mutation, a female had to hold her baby with both hands in an upright posture. In doing so, she devoted her attention persistently to the baby, strengthening mother–baby bonding. However, her activities were thereby greatly limited. She found difficulty in being suspended from branches, climbing trees, reaching high canopies to get fruits and nuts, and so on. She lived in a woodland area with patches of forest, which would prevent her from easy movement from tree to tree or canopy to canopy. In addition, she needed more nutrients than usual. The mother and baby would have starved to death if the partner male did not collect food and carry it with his hands to her. He might sometimes have taken care of the baby and strengthened family bonding. Irresponsible males could not have left their offspring. The mother would have sexually accepted him at any time as a reward for food and also desired his return to their nest. Sexual relations in a face-to-face posture, which stimulate the clitoris irrespective of the estrus cycle, might have strengthened the pair bond. Skin-to-skin contact without intervening fur might have let the pair feel deeper contact. Seasonless copulation must have caused consecutive conceptions, continuous breeding seasons, and families with members consisting of parents, a suckling baby, and junior and senior children if several years were necessary for babies to grow up. Cooperative maintenance of a family by its members must have been necessary. This would have reinforced the family bond. Consequently, the unique sexual and reproductive behavior of monomorphic humans, including monogamous and seasonless mating, lack of an externally recognizable estrous cycle, continual receptivity, and the large penis can be explained inseparably as a result of hairlessness.

Hairlessness might contribute to maintain cooperative families. Japanese macaques inhabit the Shimokita Peninsula at the northern end of the Honshu Island of Japan, the northernmost habitat of any primate in the world. Not only Shimokita macaques but others that live in Japan sometimes close together into a knot, consisting of several to more than 10 or more, to prevent heat loss in cold days. This kind of tight clustering might have occurred in hairless hominin families in cold days, provably strengthening family bond.

*Ardipithecus ramidus* had a reduced canine/premolar complex (Suwa *et al.* 2009) and showed no sexual dimorphism in body size (Lovejoy *et al.* 2009; White *et al.* 2009b), indicating less male-to-male conflict and social aggression. Granted that this is true for early hominins and that the formation of a cooperative family as the basic unit of human society occurred, no reasons exist to develop and maintain big, projecting canines and large, dimorphic male bodies. On the contrary, small canines and monomorphic bodies must have been selected. The original monogamous family system seems to have been transferred to the descendant hominins. *A. afarensis,* a plausible *Homo* ancestor, was clearly bipedal (Lovejoy 1988) and was likely to be principally monogamous, as we are (Reno *et al.* 2003).

Morris (1967) wrote that we *H. sapiens* have lived in a culturally developed society to date, but the basic unit of the society remains the family, as was true in the early hunting and gathering days. This notion must be applicable as well to early hominins. In addition, the mentality of family bonding, a sympathetic mindset trying to help and support each other substantially and mentally, is apparently expanded to human communities as human bonding. In this context, it is of interest to learn that 2- to 3-year-old human children understand collaboration and sharing, whereas chimpanzees do not (Hamann *et al.* 2011).

## Hairless mutations

Congenital disorders characterized by alterations in ectodermal structures involving alterations in hair, teeth, nails, sweat glands, cranial-facial structure, digits, and other parts of the body are known as ectodermal dysplasias (EDs). The clinical classification of EDs involves 64 genes (Visinoni *et al.* 2009). The downless (*dl*) gene mutations in mice have defective hair follicle induction, lack sweat glands, and have malformed teeth. Positional cloning of the *dl* gene has showed a novel member of the tumor necrosis factor (*Tnf*) receptor (*Tnfr*) family, of which ligand is likely to be the product of the tabby (*Ta*) gene (Headon & Overbeek 1999). This was confirmed also in the human *DL* homologue (Monreal *et al.* 1999).

Darwin (1859) alluded to the relationship between the hair and teeth in the naked Turkish dog and wrote that ‘it can be only slightly accidental’. Indeed, Mexican and Peruvian hairless dogs and Chinese crested dogs are characterized by missing hair and teeth, a phenotype called canine ectodermal dysplasia (CED). In fact, CED is inherited as a monogenic autosomal semidominant trait. A frameshift mutation in a member of the forkhead box transcription factor family (*FOXI3*) gene on the chromosome 17 was identified as the responsible gene for CED (Drögemüller *et al.* 2008). It is noteworthy that the three hairless dogs have hair on the head (Fig. 1F), as we humans do. Mexican hairless dogs have hair on the terminal area of the tail. Chinese crested dogs have long hair not only on the head but also on the tail and the lower part of the legs.

Hairless cats called Sphynx with a mutation in the *hr* gene appear to be hairless, but are actually covered with short fine lanugo hairs. A hairless chimpanzee called Cinder at Saint Louis Zoo in Missouri, USA, suffered from alopecia universalis. When she was 5 months old, Cinder began losing her hair. Her company did not discriminate against her. Unfortunately, she had no babies (http://www.ksdk.com/news/local/story.aspx?storyid=1676503). Nude mice frequently used in xenograft experiments have a mutation in the *nu* locus in the chromosome 11 and are hairless and devoid of the thymus (Takahashi *et al.* 1992). By contrast, a Burmese family included extremely hairy members and suffered from congenital hypertrichosis lanuginosa, an autosomal dominant inheritance (Bondeson & Miles 1996). They lacked teeth. This is a kind of hypertrichosis characterized by excessive growth of hair.

Some examples listed above are sufficient to understand that hairless or hairy humans are producible by a single mutation. A gene exerts its function or functions as a member of genetic networks. Thus, it sometimes or even frequently shows pleiotropic effects; effects of a mutation differ depending on the affected gene. The full set of ED genes might contain a locus associated with hairless humans with scalp hair. This mutation, probably dominant, could be accompanied by other traits in addition to hairlessness, such as modifications of dentition and sweat glands.

A recent study of human-specific deletions that led to the loss of penile spines is of interest (McLean *et al.* 2011). The authors assert that simplified penile morphology tends to be associated with monogamous reproductive strategies by the longer duration of copulation. Thus, a loss of function on the one hand is a gain of function on the other, as a hairless mutation, a loss of function, might work as a gain of function as a bipedalism inducer. In this sense, it is of importance to emphasize that analyses of speciation using 101 phylogenies of animal, plant, and fungus taxa demonstrate that 78% of the phylogenies fit the simplest model in which a new species emerged from a single, rare, stochastic event (Venditti *et al.* 2010).

## Arboreal primates were partial bipeds

Granted that humanization occurred suddenly at 6 Mya because of a single, rare, stochastic event (Venditti *et al.* 2010), that the event was a hairless mutation, and that the mutation had nothing to do with bone morphology, it follows then that bipedalism must have been partially prepared in CLCA before the mutation occurred.

Arboreal primates are not real, obligate quadrupeds. They frequently stretch their bodies in trees to reach for food such as fruits and young shoots. They hold large pieces of fruit with both hands and eat them although sitting on a branch or cliff or on the ground. They sometimes, so to speak, ‘walk’ on branches using a hand or both hands for support. Gibbons actually walk or run on branches in jungles or walk or hop on a tightrope in the zoo. Japanese macaques sometimes show upright walking to carry food such as sweet potatoes, which is washed using both hands. Some bathe in hot springs in winter in a human-like posture. They can be trained to walk as bipeds for human entertainment. Orangutans, so-called men of the forest, can walk upright in the trees of jungles or skywalk in zoos (Fig. 1A) as can chimpanzees (Fig. 1B). Not only arboreal primates but also terrestrial ones such as baboons are not obligate quadrupeds; they pick up food using a hand and eat it in a sitting posture on the ground or on a cliff. When they wade in shallow streams, they walk upright or hop. The scarcity of these examples from a large collection of bipedal records observed throughout primates suggests that CLCA was not an obligate quadruped, but a partial biped as other arboreal and terrestrial primates are. Consequently, a hairless mutation was likely to have forced human ancestors to become facultative bipeds from partial bipeds. The idea that ‘emergent hominids were predisposed to terrestrial bipedalism by a heritage of bipedal and other orthograde positional behavior in trees (Tuttle 1975)’ must be correct.

## Disadvantageous traits of bipedalism and hairlessness

Granted that all primates were partial bipeds and that hairless mutations were able to occur evenly among them, why did only humans become concomitantly bipedal and hairless? The answer might lie in the disadvantageous traits of bipedalism and hairlessness, both of which could have been narrowly overcome through family bonding.

Bipedalism is apparently not advantageous because bipedal runners use a greater volume of muscle to support their body weight (Roberts *et al.* 1998). Variations in the efficiency of human locomotion appear to be similar to those of terrestrial quadrupeds at best (Steudel-Numbers & Wall-Scheffler 2009). The animal kingdom’s champion runner, the cheetah, can run at 30 m/s, whereas a human sprinter runs at 10 m/s. Some other examples are as follows: the leopard, 16; the wolf, 17; the dog, 18; and the gorilla 8 m/s (Iriarte-Díaz 2002). This simple, symbolic comparison suggests that quadrupedalism is superior to bipedalism from the viewpoint of speed running. To pursue prey and to escape from predators, a high-speed running ability must have been necessary for early hominins to the degree that they tried to live in a terrestrial setting. Hairless human ancestors must not have been bestowed with such running ability; they would have remained in a woodland setting, where they found food, slept, and avoided predators. As facultative bipeds, their movement must not have been so much swifter than that of other partial bipedal primates. Modern persons might be good endurance runners, but this must have been achieved at the *Homo* stage (Bramble *et al.* 2004). Consequently, bipedalism must have been disadvantageous not only for arboreal denizens but also for terrestrial denizens. Actually, bipedalism must have been selected against in all primates except humans.

Fur protects hairy animals from strong sunlight and from UV in particular. Furthermore, it is useful to keep their bodies warm in cool ambient temperatures, which is not rare even in savannahs. It must also be useful to protect their naked skins, which are vulnerable to injury. Most importantly, body hair is indispensable for primates to carry babies (Fig. 1A–D). Thus, all hairless primates except for hominins, if so-born, must have been selected against, and all primates excepting humans are hairy now even in hot areas. Consequently, human ancestors with a hairless mutation had the negatively synergistic burdens of disadvantageous bipedalism and hairlessness. They must not be easily allowed to access fertile areas and be forced to live difficult lives under adverse conditions. They must survive the difficulties on the verge of extinction for a very long period, until genetic and epigenetic changes in addition to environmental changes spurred the additional evolutional selection of humans after the first hairless mutation in CLCA. Only those who entered through the narrow gate and survived trials and tribulations in cooperation with family members were able to survive in a new world by making the most of their free hands, and later, by finding some advantages of brain enlargement, which was facilitated by bipedalism. This is reminiscent of the formula learned in elementary school, by which a minus multiplied by a minus produces a plus.

## Sweat gland and breast development and hairlessness

Common involvement of a single gene in the formation of human teeth, hair follicles, sweat glands, and breasts is a legacy of the evolutionary history that these organs share. The fur-lacking human epidermis has 2–5 million eccrine sweat glands and produces up to 12 L of sweat daily (Jablonski 2010). Hairlessness might have contributed to the development of the large number of eccrine glands by providing the skin with sufficient room for them.

The hair follicle is a simple duct. The breast consists of a bulging burden of ducts. As with the eccrine glands, a hairless mutation might have provided early hominins with a predisposition to unique human breasts, which present a sharp contrast with those of monkeys and great apes in that they protrude prominently from the smooth, hairless chest with the nipple in the center of the areola (Fig. 1E), even during a nonlactating period. The constant exhibition of enlarged breasts in a female can be explained by seasonless mating, consecutive conception, and continuous breeding. Round enlarged breasts in an upright female might be accepted delightedly by an upright male as symbolizing fecundity (Fig. 1E). Even after bipedalism was established, the charming breasts were most probably effective at maintaining hairlessness positively.

## Conclusion

A single hairless mutation could compatibly and inseparably explain three major characteristics that distinguish humans from other primates, bipedality, practical nakedness, and the family as a social unit. A newborn or infant baby of all primates except humans clings to the mother by grasping her hair firmly, whereas a human baby has nothing to grasp; the mother must hold the baby with both hands, enforcing upright walking. In ED, a semidominant single mutation is known to induce hairless animals with scalp hair. A hairless mutation must have played a crucial role in the process of humanization from a human–ape common ancestor.
